# Supplementary material for: A high-throughput integrated biofilm-on-a-chip platform for the investigation of combinatory physicochemical responses to chemical and fluid shear stress
Source: PLoS One. 2022 Aug 12;17(8):e0272294. doi: 10.1371/journal.pone.0272294 (PMC9374262; doi:10.1371/journal.pone.0272294)
Supplement: S1 File — (DOCX) [file pone.0272294.s001.docx]

**Supplementary Material**

**A high-throughput integrated biofilm-on-a-chip platform for the investigation of combinatory physicochemical responses to chemical and fluid shear stress**

Ann V. Nguyen^1*^, Arash Yahyazadeh Shourabi^1*^, Mohammad Yaghoobi^1^, Shiying Zhang^2^, Kenneth W. Simpson^2^, Alireza Abbaspourrad^1^

^1^*Department of Food Science, College of Agricultural and Life Sciences, Cornell University, Stocking Hall, Ithaca, NY, 14853*

^2^*Department of Clinical Sciences, College of Veterinary Medicine, Cornell University, 602 Tower Rd., Ithaca, NY, 14853*

^*^*These authors contributed equally*

**Corresponding author: Alireza Abbaspourrad**

**Email:** Alireza@cornell.edu

**Tel.:** (607) 255-2923

**Address:** 243 Stocking Hall, Ithaca, NY 14850-7201


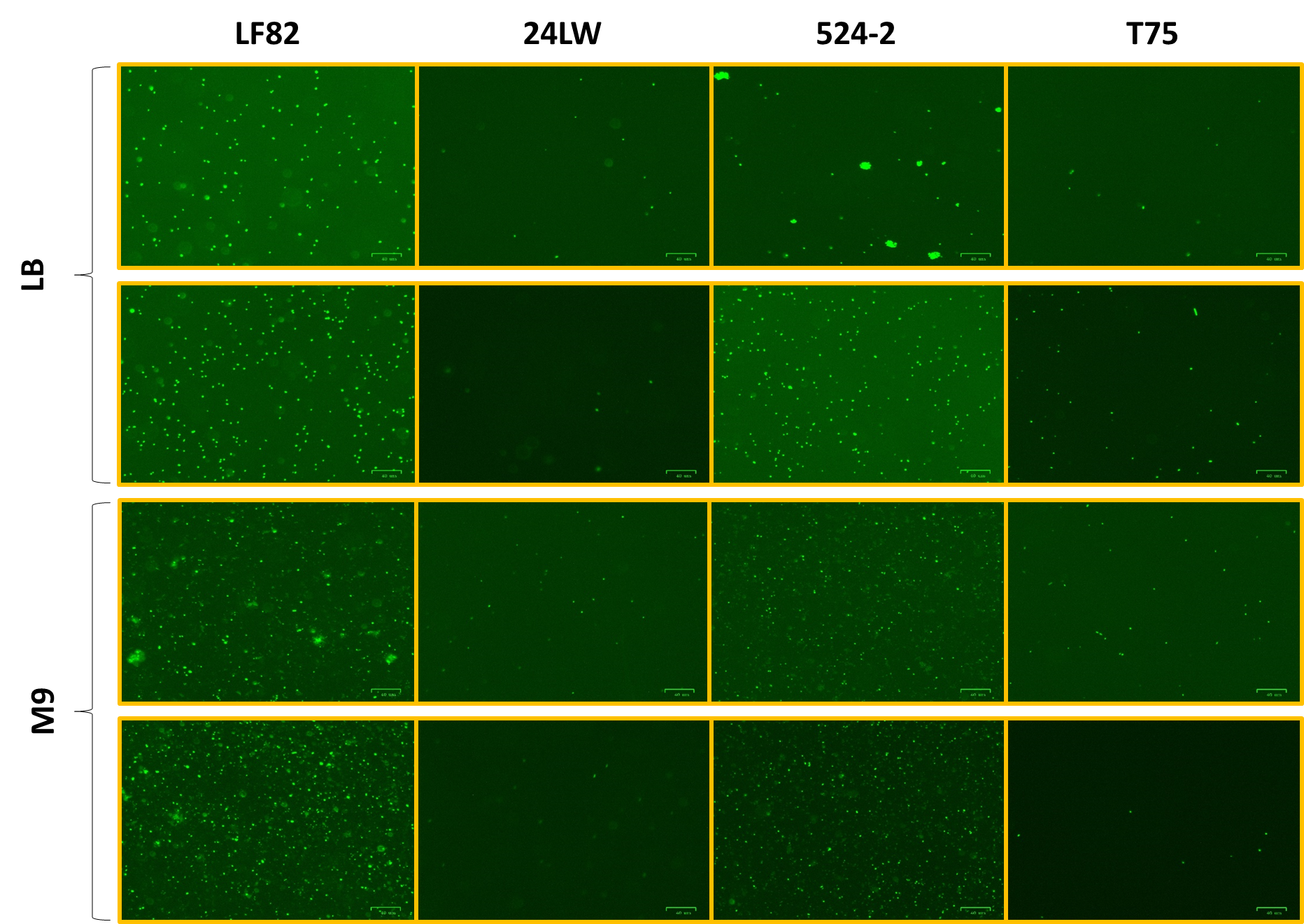


Supplementary Figure S1. Surface attachment and biofilm formation screening of four *E. coli* strains in LB and M9 culture media after 24 hr incubation. Study was conducted in 96-well plate at static condition.


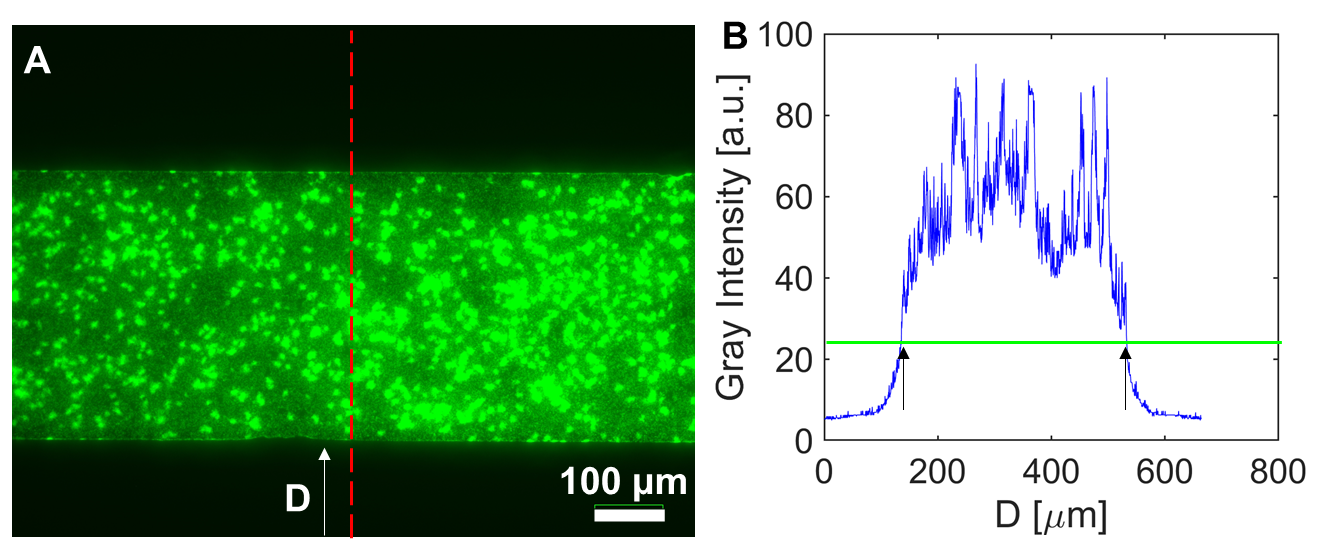
Supplementary Figure S2. Images of the channel with biofilms were scanned and the threshold of 25/255 were selected to identify the walls of the channel. We scan from black area to black area (top to bottom of the picture) and then comparing the image of the channel with the graph of the fluorescence intensity we observed two things: first there is a halo just outside of the channel wall and secondly that the intensity increases sharply in the graph when the scan hits the true channel. The halo causes the intensity to rise slightly as we approach the channel until a point where it is clear we’ve hit the channel where we observe a strong signal change. We found that 25/255 was similar on both sides of the channel and represents the halo that is observable in our image. In addition, we were able to use the width of the channel to double check our observations. That is: we identified the region of the graph that corresponds to the channel, which is the region between the two arrows on our graph. The background does not vary along the channel, and this is true for images of the medium and high FSS zones where the widths were 400 and 100 µm, respectively. The low FSS zone did not include the channel walls, because the width of the zone was larger than the field of view.


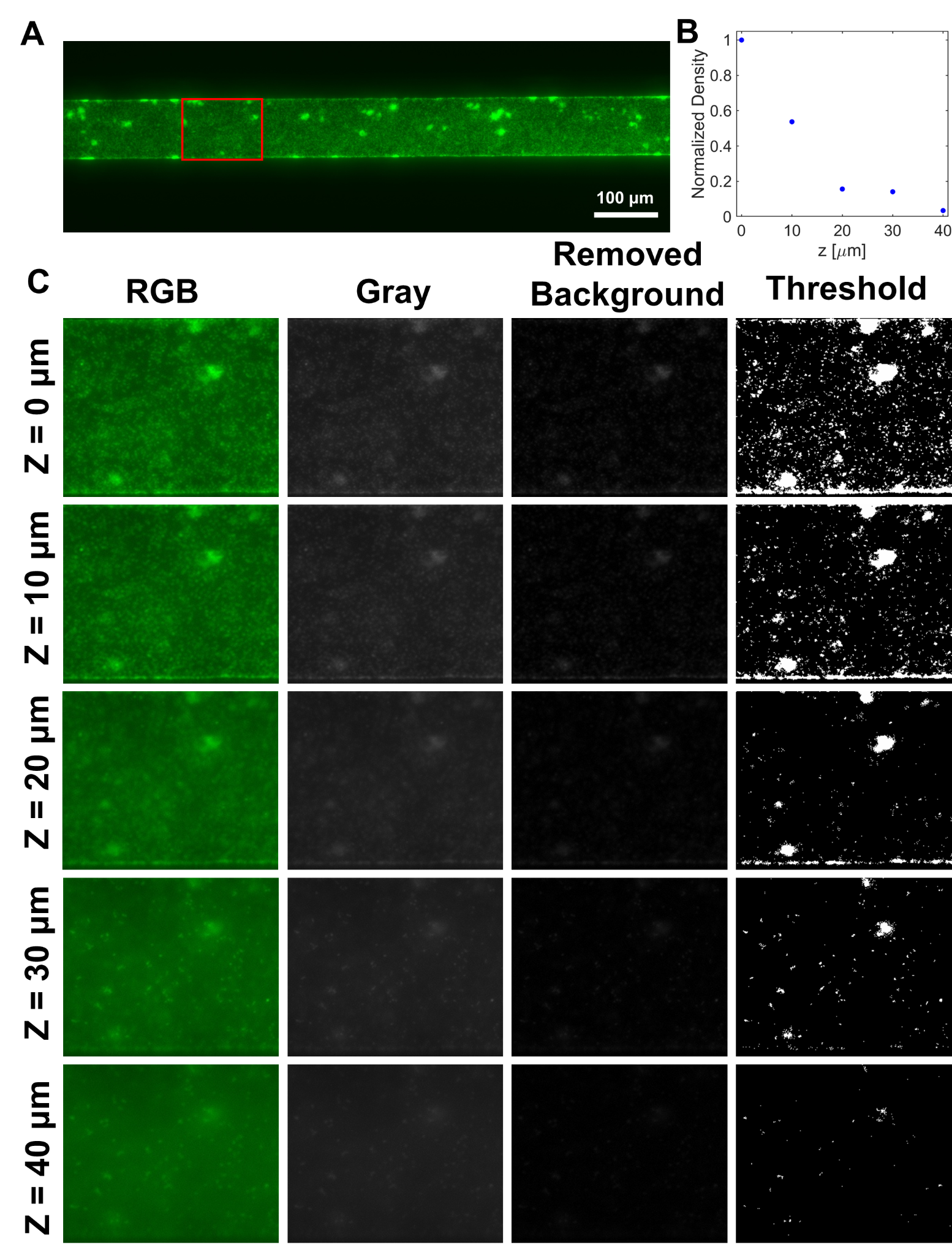


Supplementary Figure S3. Examining effect of out-of-focus signal. Fluorescent images of the channels with biofilm A) are taken at the interval of 10 microns from the bottom to the top. The bottom was defined as z = 0 um. Then they were converted to gray scale images. Then the background of the images are removed using the ImageJ utilizing background subtraction code. Then using a threshold value of 50, the images are binarized. Using ImageJ’s analyze particle plugin, the particles with size of more than 2 pixels were counted and the number of particles are normalized with the maximum amount found in the lower wall. The data is shown in part B of this figure and shows how the number of bacteria in the images decreases drastically from 10 to twenty microns. Except for those large clumps of mass of bacteria in the lower wall, the majority of the signal from the bottom does not reach the top wall and vice versa. In summary, the majority of the signal is coming from the lower wall which is detected in the images, and therefore various cases in this study could be compared with each other regarding the total fluorescent intensity and the surface coverage.


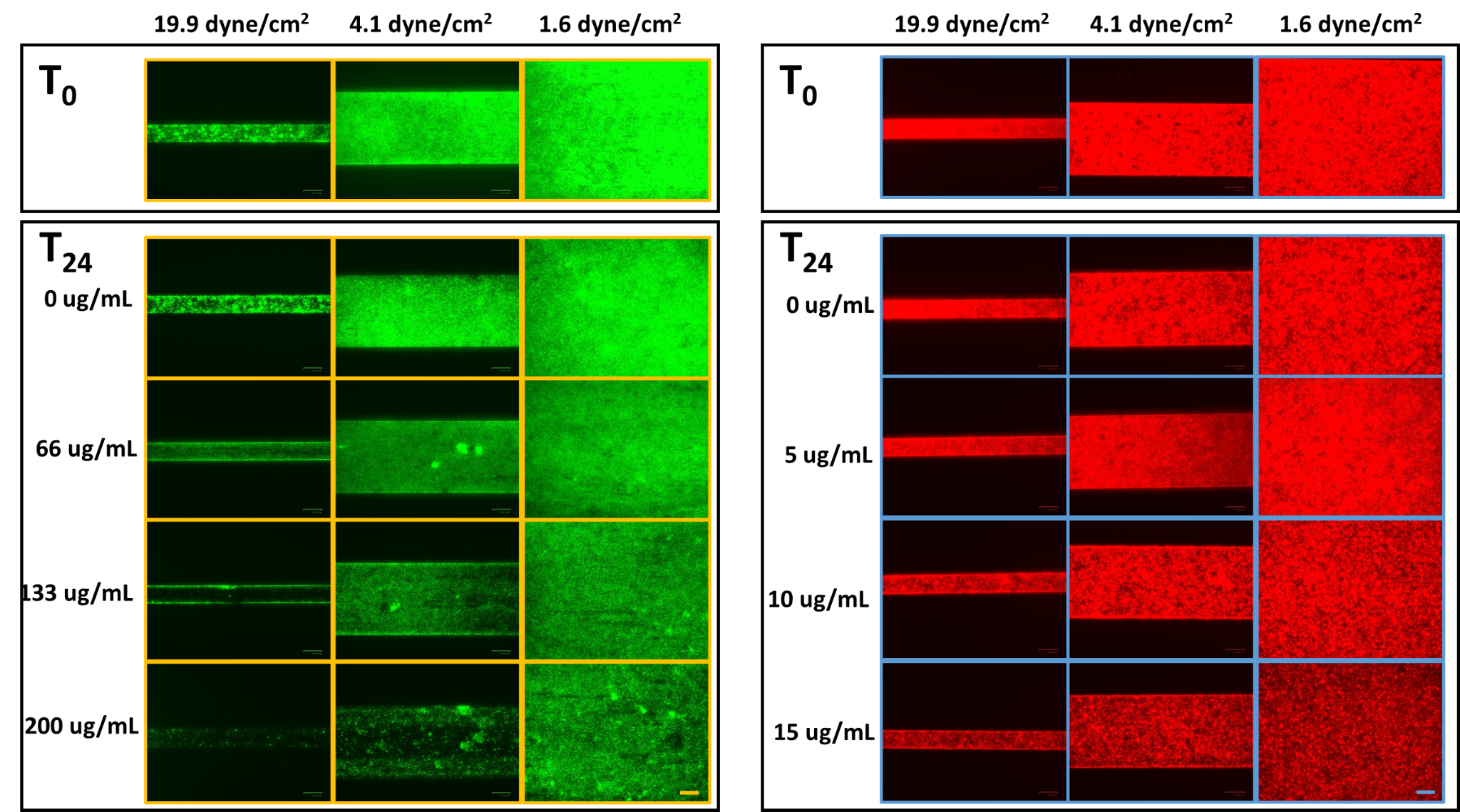


Supplementary Figure S4. Fluorescent images of the on-chip A) *E. coli* LF82 biofilms and the physicochemical effects of increasing concentration of streptomycin and fluid shear stress after 24 h treatment. B) Effects of gentamicin and fluid shear stress on *P. aeruginosa* biofilms showing changes with increasing antibiotic concentration and shear stress. No significant changes were observed when treated with streptomycin (data not shown for lack of effect). Scale bar = 100µm
