# Supplementary material for: A high-throughput integrated biofilm-on-a-chip platform for the investigation of combinatory physicochemical responses to chemical and fluid shear stress
Source: PLoS One. 2022 Aug 12;17(8):e0272294. doi: 10.1371/journal.pone.0272294 (PMC9374262; doi:10.1371/journal.pone.0272294)
Supplement: Attachment — Submitted filename: KJD 7.12.22. Biofilms Response.docx [file pone.0272294.s002.docx]

**Response to Reviewers**

The authors thank the reviewers for their time and careful review of our work. We have used the comments to improve the clarity of our manuscript and have made modifications as recommended by the reviewers. The authors’ responses to the comments raised by reviewers are listed below, and changes have been tracked in the manuscript.

**Reviewer #1:**

This is a very interesting piece of work that describes a very promising technique for the study of biofilm eradication. I am not qualified to make a judgement on the fluid dynamics of the device. However, I do have a issues with the methodology. I think that if they are addressed, then the to manuscript will be greatly improved:

1) I cannot see a description of the strain of P. aeruginosa. Why was the particular strain chosen? This must be included in the Experimental section.

Thank you for pointing this out. *Pseudomonas aeruginosa* is a pathogenic bacteria that can cause infections in humans, mostly in hospital patients. It forms antibiotic-resistant biofilms that make it difficult to eradicate. Thus, it was considered a good model system for our study. Since it is easier to monitor the changes of biofilms with fluorescent-labeled bacteria, we chose to use a RFP labeled *P. aeruginosa* that was transformed with RFP-plasmid carrying ampicillin resistant gene. We used PA01, a common lab strain that is kept and maintained as a part of the Simpson Lab collection.

The description was added to the revised manuscript.

2) The choice of PDMS for the device is very interesting. The authors should describe the choice of PDMS in more detail, highlighting its use in current medical devices, e.g. catheters. What are the properties of this material with regard to bacterial adhesion and growth? Do the bacteria adhere mainly to the glass bottom layer or the PDMS? If to the glass, what is the relevance with regard to the clinical situation that the authors appear to be addressing in this application of the device?

Thank you for suggesting that we should address this. The choice of PDMS for the device guarantees its universality for a broad range of applications since the stiffness is tunable mechanically even several orders of magnitude. Consequently, to mimic the specific environment where the biofilm of interest grows, the operator can simply tune the stiffness of the PDMS substrate by changing the ratio of PDMS to cross-linker or the baking time and temperature. For the simplicity of this proof-of-concept study, we used a standardized PDMS mixing ratio and curing condition, but future studies can further explore the effect of surface stiffness on biofilm eradication. Choosing PDMS is also advantageous as the device can be used for investigating silicon coating materials for a broad range of implantation applications. PDMS is widely used as a coating material in neural and cochlear implants^[1]^. PDMS-based biomaterials are also used for catheter fabrication with antimicrobial properties^[2]^. In this work all the bacteria is adhered to the PDMS layer which is bonded on the glass slide. PDMS is the substrate for the formed biofilm. Stiffness of PDMS does not affect the bacterial adhesion, however, it controls bacterial retention^1^ which was not the goal of this study.

This discussion was added to the conclusion in the revised manuscript and these are references 40 and 52 in the main manuscript..

3) A MIC is performed but an MBEC (i.e. biofilm eradication) under standard conditions would have been very useful, to compare the type of data most often obtained with the data obtained using the authors' device.

Per the reviewer’s suggestion, we examined the MBEC for these bacteria/antibiotic combinations using commercially available system (MBEC Assay kit, Inovotech) and found that the MBEC values by this conventional method reflect the findings in treatments at the lowest fluid shear stress (1.6 dyne/cm^2^) in our device. The MBEC values of *E. coli* treated with gentamicin and streptomycin were 10 and 200 µg/mL, and the values of *P. aeruginosa* treated with these antibiotics were 20 and 400 µg/mL, respectively. While a useful comparison, these values, however, reflect different phenomena, specifically where the commercial assay calls for sonication to disrupt the remaining biofilm after an antibiotic challenge, whereas our system quantifies based on the remaining intact biofilm on the surface. Additionally, while the conventional method focuses more on antibiotic penetration and killing of the bacteria within the biofilm, our method allows further examination of other properties of the biofilm such as the ability to resist shear stress or adhesion/detachment from the surface, which could be useful in some applications to remove the biofilm.

The experiment and results were added to the material and method, and the result and discussion sections were revised.

4) It is unclear from the Experimental as to exactly how images were obtained. What type of micrscope was employed? Was a z-stack made after locating the upper and lower walls of the channel? If not, how do the authors' guarantee that the fluorescence truly represents the three-dimensional array of bacteria? Was the depth of field somehow large enough to have the whole of the biofilm in focus? How does out-of-focus fluorescence register compared to in-focus? Is there a bias when measuring a thicker biofilm compared to a thinner one?

These are great questions, thank you for asking it. The images obtained for quantitative analysis of the treatments were taken using an inverted microscope (Bio-rad ZOE Fluorescent Cell Imager), and a z-stack was not made. A z-stack would place more emphasis on biology where the scope of this work was intended to place more focus on the chip design. Instead, we only focus on the two-dimensional array of biofilms at the bottom wall. We have included an additional figure in the supplementary (S3) showing the changes in the fluorescent signal as the focus moves from the lower to the upper wall (z(0) to z(40) in 10 µm increments). There are some minor bacterial attachments on the upper wall that may contribute to off-target fluorescence signals. However, for both green (*E. coli*) and red (*P. aeruginosa*) biofilms, the out-of-focus light intensity contribution to the in-focus fluorescence intensity decreases as we focus on the lower wall (which contains the majority of the biofilm intensity). The images show that the signal is focused and specific at the z(0) focal plane, which is what we focus on to take the 2D images of the biofilm’s surface. And because the two-dimensional imaging at this z-axis position is mainly in-focus signal, we hypothesize that a thicker biofilm might not cause a bias in this study.

We agree with the reviewer that the total fluorescent intensity might not truly represent the total three-dimensional array and the biomass. However, we hypothesize that the changes in total fluorescent intensity represents a net change in the biofilm. This change might be the result of phenomena including bacterial death, reduction of the biofilm thickness, detachment from the substrate surface, or their combinations. We are currently working on more in depth characterization of the on-chip biofilms using methods such as confocal microscopy and scanning electron microscopy which can help us better breakdown the bulk changes that we have observed in this study. While these improvements are underway, the focus of this work was proving that we could measure the combined impact of antibiotics and shear on biofilms. Further studies will refine many aspects of this platform.

5) How was the background fluorescence of 25/255 calculated? Did background not vary at all in different channels?

Thank you for your comment. We have added supplementary figure S2 to illustrate why an intensity of 25 was chosen for the background. We scan from black area to black area (top to bottom of the picture) and then comparing the image of the channel with the graph of the fluorescence intensity we observed two things: first there is a halo just outside of the channel wall and secondly that the intensity increases sharply in the graph when the scan hits the true channel. The halo causes the intensity to rise slightly as we approach the channel until a point where it is clear we’ve hit the channel where we observe a strong signal change. We found that 25/255 was similar on both sides of the channel and represents the halo that is observable in our image. In addition, we were able to use the width of the channel to double check our observations. That is.we identified the region of the graph that corresponds to the channel, which is the region between the two arrows on our graph. The background does not vary along the channel and this is true for images of the medium and high FSS zones where the widths were 400 and 100 µm, respectively. The low FSS zone did not include the channel walls, because the width of the zone was larger than the field of view.

6) It is unclear in Figure 1 as to where the microscope is positioned - can this be clarified? In addition, what magnification was used? Was the whole 'zone' of each channel imaged just once at each time point?

Thank you. We modified Figure 1 to clarify the microscope position. We used the Bio-rad ZOE Fluorescent Cell Imager which has one 20x objective lens and 175x display magnification. No additional magnification was used. The whole zone was twice the length of the field of view, so we took two images to get the whole length of every zone (three zones in a channel) at every time point.

Additional clarification was added to the materials and method section.

7) It would be very helpful to include an indication of the statistical significances observed in Table 1 (or as a separate Table, if necessary), rather than merely mentioning some examples in the text.

Thank you. Table 1 has been revised accordingly to clarify statistical significance.

8) The authors conclude that the different susceptibilities of biofilms to antibiotics is a result mainly of drug penetration etc due to 'the difference in EPS composition and physicochemical characteristics'. An alternative explanation might be the response of P. aeruginosa to shear stress ( doi: 10.1016/j.bpj.2010.11.078). Increasing adhesion of the biofilm to the substrate might counteract the effects of the antibiotics.

Thank you for the suggestion. We added this point and the reference to the discussion.

**Reviewer #2:** 

The manuscript describes the use of integrated double-layered microfluidic chip for assessing the physiochemical responses of bacterial biofilms or to assess the effectiveness of biofilm removal methods. Yet, it requires strenuous experimental validation to prove the same.

Major comments
1. The proof-of-concept study revealed the potential of combinatorial effect of antibiotics and fluid shear stress. Yet, the study is limited to specific bacterial pathogens and antibiotics. As a proof-of-concept study, studying its effect on different pathogens and with last resort antibiotics is much needed. More, the feasibility of this chip-based high-throughput screening method should be vigourously discussed based on the results obtained from the results.

We thank the reviewer for the constructive criticism. We believe that the platform can accommodate many more pathogens and antibiotics beside the ones used in this proof-of-concept feasibility study. To allow clinical relevance of the findings, we considered bacteria that have a history of developing biofilms and that can cause infections in humans as our model systems. Especially *P. aeruginosa*, which forms antibiotic-resistant biofilms that make it difficult to eradicate.

The high-throughput screening ability of the platform can be improved by adding more FSS zones and/or having larger CGG to achieve a broader range of FSS and more increments of both FSS levels and antibiotic concentrations. Here, we demonstrated only 12 combinations (4 concentrations vs. 3 shear stress levels), but the platform can be designed to include a higher number of factors ( for example, 12 concentrations vs. 12 shear stress levels) using the same design and optimization principles. The platform can offer testing at as many concentration levels as a commercially available system based on a 96-well plate, with the addition of fluid shear stress factor.

We have added this discussion to the conclusion section in the revised manuscript.

2. Showing the biofilm forming ability of the test pathogen used in this study as supplement would able the readers to interpret the data.

Thank you for asking this question. While we did not conduct an in-house study of our pathogens’ ability to form biofilms, results previously published have suggested that they are both strong biofilm formers based on their Specific biofilm formation (SBF) index. The SBF index of *E. coli* LF82 was reported to be 1.6 to 2, and of *P. aeruginosa* PA01 was reported to be > 3.5. Bacteria with SBF >1 are considered to be strong biofilm producers.

Additionally, we used a standard method for determining MBEC with our bacteria to compare with our on-chip results. We have added this test as well as the following references to the paper.

Martinez-Medina, M., Naves, P., Blanco, J., Aldeguer, X., Blanco, J. E., Blanco, M., ... & Garcia-Gil, L. J. (2009). Biofilm formation as a novel phenotypic feature of adherent-invasive Escherichia coli (AIEC). *BMC microbiology*, *9*(1), 1-16.

Sicard, J. F., Vogeleer, P., Le Bihan, G., Rodriguez Olivera, Y., Beaudry, F., Jacques, M., & Harel, J. (2018). N-Acetyl-glucosamine influences the biofilm formation of Escherichia coli. *Gut pathogens*, *10*(1), 1-10.

Kiran, S., Sharma, P., Harjai, K., & Capalash, N. (2011). Enzymatic quorum quenching increases antibiotic susceptibility of multidrug resistant Pseudomonas aeruginosa. *Iranian Journal of Microbiology*, *3*(1), 1.

3. Like figure 4 and supplemental file, showing the data for biofilm eradication of Pseudomonas aeruginosa (fluorescent images) in the presence of FSS and antibiotics would be better as supplement.

Thank you. We have created a figure similar to Figure 4 for *P. Aeruginosa* biofilm and added it to the supplementary material.

4. Page 16, the MIC of gentamycin (2 ug/ml) and streptomycin (25 ug/ml) was same for both the pathogen. For the study using fluidic chip, the concentration used were 15 and 200 ug/ml, which is more than 10-fold higher than the MIC. Justify?

Thank you for asking for more information here. We expected the minimal biofilm eradication concentration (MBEC) to be much higher than the MIC, as described in the manuscript that “the required dosage of an antibiotic drug to kill bacteria in a biofilm is usually higher than the concentration needed to kill them in the planktonic phase.” Therefore, we initially screened for a broad range of concentrations 5-10 times the MICs. We picked the target concentrations to best demonstrate the species-specificity and antibiotic-specificity in this assay. The results from the MBEC assay using commercial kits also support our testing range. We found that the MBEC values by this conventional method reflect the findings in treatments at the lowest fluid shear stress (1.6 dyne/cm^2^) in our device. The MBEC values of *E. coli* treated with gentamicin and streptomycin were 10 and 200 µg/mL, and the values of *P. aeruginosa* treated with these antibiotics were 20 and 400 µg/mL, respectively.

We have added language to this effect in our revised manuscript.

6. PLOS authors have the option to publish the peer review history of their article ([what does this mean?](https://journals.plos.org/plosone/s/editorial-and-peer-review-process#loc-peer-review-history)). If published, this will include your full peer review and any attached files.


If you choose “no”, your identity will remain anonymous but your review may still be made public.


**Do you want your identity to be public for this peer review?** For information about this choice, including consent withdrawal, please see our [Privacy Policy](https://www.plos.org/privacy-policy).

Reviewer #1: **Yes:** Peter Monk

Reviewer #2: No

1. [Siddiqui, S.; Chandrasekaran, A.; Lin, N.; Tufenkji, N.; Moraes, C. Microfluidic Shear Assay to Distinguish between Bacterial Adhesion and Attachment Strength on Stiffness-Tunable Silicone Substrates. *Langmuir* **2019**, *35* (26), 8840–8849.](https://www.zotero.org/google-docs/?OKpqdb)
2. [Armugam, A.; Teong, S. P.; Lim, D. S.; Chan, S. P.; Yi, G.; Yew, D. S.; Beh, C. W.; Zhang, Y. Broad Spectrum Antimicrobial PDMS-Based Biomaterial for Catheter Fabrication. *Biomaterials research* **2021**, *25* (1), 1–13.](https://www.zotero.org/google-docs/?OKpqdb)
